# Supplementary material for: Inhibition of Clostridium difficile TcdA and TcdB toxins with transition state analogues
Source: Nat Commun. 2021 Nov 1;12:6285. doi: 10.1038/s41467-021-26580-6 (PMC8560925; doi:10.1038/s41467-021-26580-6)
Supplement: Supplementary file 1 — Supplementary Information [file 41467_2021_26580_MOESM1_ESM.pdf]

**Inhibition of *Clostridium difficile* TcdA and TcdB toxins with transition state analogues**

Ashleigh S. Paparella[1]., Briana L. Aboulache[1#]., Rajesh K. Harijan[1]., Kathryn S. Potts[2] Peter C. Tyler[3] and Vern L. Schramm[1*].

1. Department of Biochemistry, Albert Einstein College of Medicine, Bronx, New York, USA
2. Department of Developmental and Molecular Biology and Gottesman Institute of Stem Cell Biology and Regenerative Medicine, Albert Einstein College of Medicine, Bronx, New York, USA
3. The Ferrier Research Institute, Victoria University of Wellington, Lower Hutt, New Zealand

#Present address: Department of Biochemistry, University of Colorado Boulder, Boulder, Colorado, USA.

*Address correspondence to: Vern L. Schramm, Golding building Rm 301, Department of Biochemistry, Albert Einstein College of Medicine, 1300 Morris Park Avenue, Bronx, NY, 10461. Phone: 718-430-2813, Email: vern.schramm@einsteinmed.org

## Supplementary Information

A. **Supplementary Methods**
B. **Supplementary Figure 1-12**
C. **Supplementary Table 1**
D. **Supplementary References**

## A. Supplementary Methods

### Synthesis of isofagomine

Isofagomine was synthesized as reported (Rybczynski, P.; Tretyakov, A.; Fuerst, D.; Sheth, K. New method for preparing isofagomine and it's derivatives, US Patent 2010/0160638) with some modifications. Nitrile **1** (prepared from D-arabinose) was reduced with $BH_3.SMe_2$ to the amine which was isolated as it's N-Boc derivative **2**. This was then treated with aq HCl followed by hydrogenolysis affording isofagomine **3**.

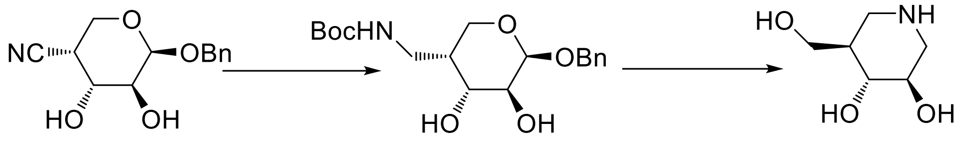


1 2 3

A solution of **1** (0.507 g, 2.03 mmol) in dry THF (10 mL) was stirred at RT under argon and borane dimethyl sulphide complex (1.03 mL, 0.822 g, 10.2 mmol) was added, then the solution was heated under reflux for 16 h. Methanol (8 mL) was carefully added and the solution was heated under reflux for 3 h and then concentrated to dryness. Methanol was added to the residue and the mixture concentrated to dryness three times. Then methanol (20 mL) was added followed by 10% aq sodium carbonate solution (5 mL). Di-tert-butyl dicarbonate 1.35 g, 6.10 mmol) was added and the mixture was stirred for 1 h and then concentrated to dryness. Chloroform (30 mL) was added and the mixture was filtered through celite and the solids washed with chloroform. Evaporation of the filtrates gave a solid. This was triturated with hexanes, filtered and dried to give **2** (0.62 g, 1.75 mmol, 86%) with NMR spectra consistent with that reported[1].

To a solution of **2** (9.80 g, 27.7 mmol) in THF (100 mL) was added 5M aq HCl (75 mL) and the solution was stirred at RT for 2 h and then concentrated to dryness. A solution of the residue in methanol was treated with Amberlyst A26 (OH-) resin until the solution pH was > 7, and then filtered and concentrated to dryness. The residue in THF (100 mL) and methanol (100 mL) was stirred under hydrogen in the presence of 10% Pd/C for 16 h. After removal of the solids and solvent and chromatography of the residue DCM/MeOH/aq $NH_3$ 5:4:1) afforded isofagomine **3** (2.80 g, 19.0 mmol, 68%) which could be converted to the tartrate salt as reported[1].

**Synthesis of Noeuromycin.**

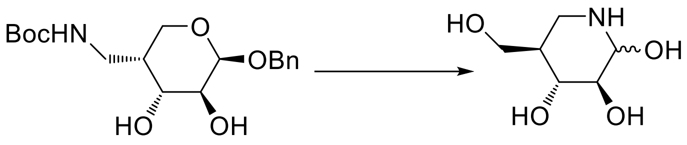


**2** **4**

A solution of **2** (115 mg, 0.33 mmol.) in 50% aq THF (16 mL) was stirred with 10% Pd/C under a hydrogen atmosphere for 16 h. Then the solids and solvent were removed. Chromatography (1-6% MeOH/EtOAc) gave the debenzylated intermediate (77 mg). This material was dissolved in 1M aq HCl (5 mL) and after 2 h, the solution was concentrated to dryness affording **4** (50 mg, 0.25 mmol, 75%) with NMR spectra as reported[2].

## B. Supplementary Figures 1-12

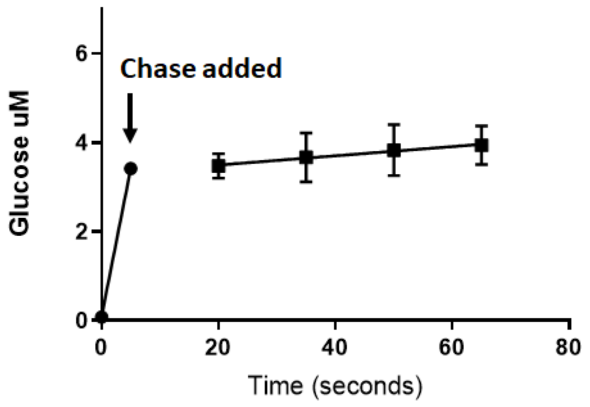


**Supplementary Figure 1. Measurement of forward commitment ($C_f$) for UDP-glucose hydrolysis with TcdB-GTD.** TcdB-GTD was mixed with [6″-$^{14}$C]-UDP-glucose and incubated for 5 seconds to form 3.4 µM glucose. At that time the reaction was chased with a large excess of unlabeled UDP-glucose and the synthesis of additional glucose after the chase was monitored to 65 sec. After extrapolation to the origin [glucose] = 3.29 ± 0.04 µM; using eq 8, 9 and 10 the $C_f$ determined was 0.064 ± 0.01. Data was plotted using GraphPad Prism 8. Data represents the mean of each time point and error bars represent ± SEM. Source data provided as a Source Data file.

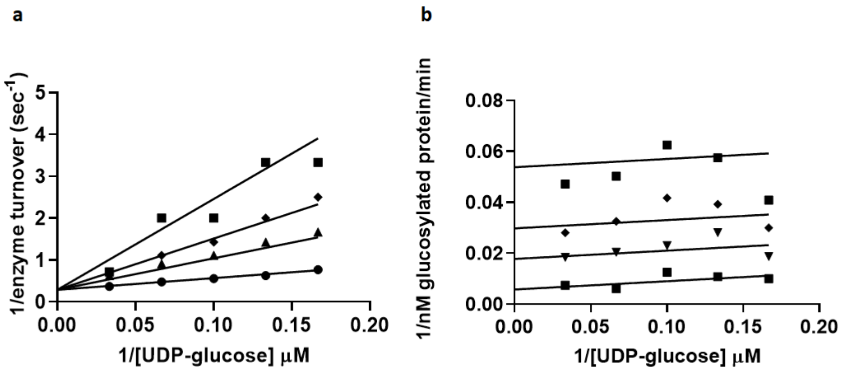


**Supplementary Figure 2. Kinetic analysis of Inhibitor binding. a** TcdB-GTD glucosyltransferase activity was measured in the presence of UDP and varying concentrations of UDP-glucose. Lineweaver Burk curves are shown for 6-$^3$H UDP-glucose. Squares represent 40 µM UDP, diamonds represent 20 µM UDP, triangles represent 10 µM UDP and circles represent 0 µM UDP. Data points are the mean of experimental data and lines represent the global fit to equation 13 for competitive inhibition as described in methods. **b** TcdA-GTD glucosyltransferase activity was measured in the presence of isofagomine and varying concentrations of UDP-glucose. Squares represent 4 µM isofagomine, diamonds represent 2 µM isofagomine, inverted triangles represent 1 µM isofagomine and squares represent 0 µM isofagomine. Data points are the mean of experimental data and lines represent the global fit to equation 12 for uncompetitive inhibition as described in methods. Source data provided as a Source Data file.

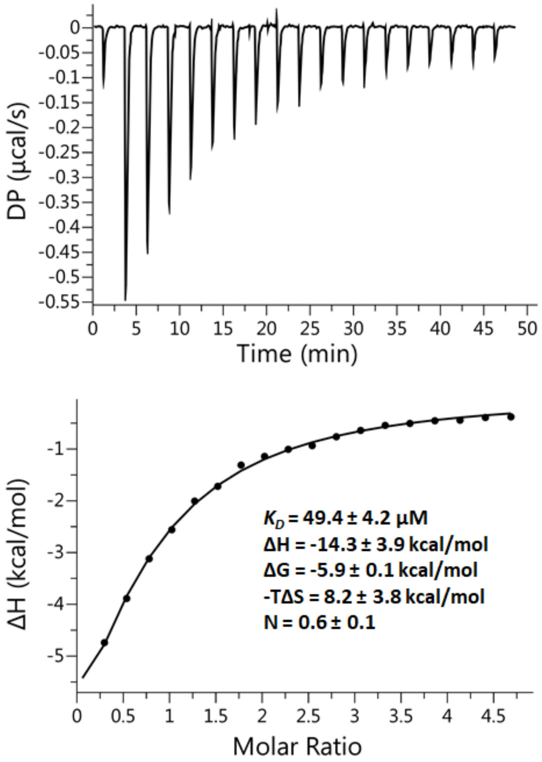


**Supplementary Figure 3. ITC titration of UDP binding to TcdB-GTD.** UDP (1 mM) was prepared in reaction buffer (50 mM HEPES pH 7.5, 100 mM KCl, 4 mM $MgCl_2$, 1 mM $MnCl_2$) titrated into a solution containing reaction buffer and 40 µM TcdB-GTD. Data was fitted using a single binding site model. Source data provided as a Source Data file.

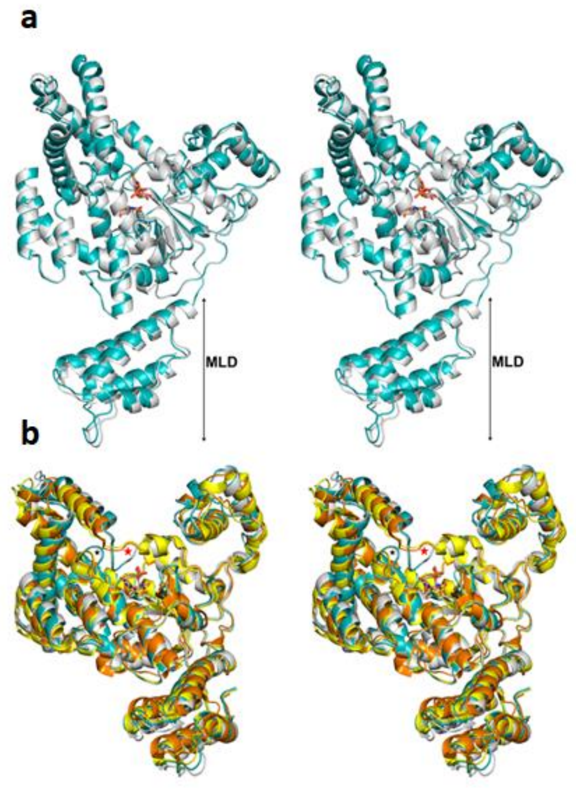


**Supplementary Figure 4. Structures of TcdB-GTD (stereoviews) in complexes with isofagomine and noeuromycin**. **a** Superposition and overall structural fold of TcdB-GTD with UDP and isofagomine (cyan) or noeuromycin (gray). The membrane localization domain (MLD) of the structure is highlighted. **b** Superposition of apo TcdA-GTD (orange, PDB ID: 3SS1), apo TcdB-GTD (yellow, PDB ID: 5UQT), TcdB-GTD with UDP and isofagomine (PDB ID: 7LOU, cyan) or noeuromycin (PDB ID: 7LOV, gray). Binding of inhibitors closes two loops from residues Glu449 to Asp461 (2.34 Å, highlighted with a black asterisk) and from Gln510 to Asp523 (10 Å, highlighted with a red asterisk) in the binding pocket.

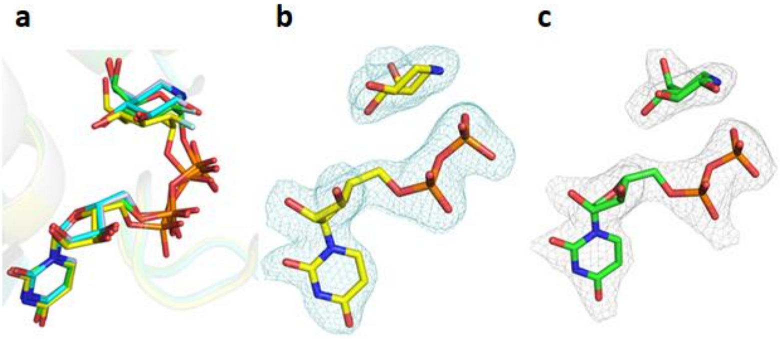


**Supplementary Figure 5. a** Ligand superposition of TcdA-GTD complex with UDP-2F-glucose (green, PDB ID: 5UQL), TcdB-GTD complex with UDP-2F-glucose (yellow, PDB ID: 5UQN), and TcdB-GTD in complex with UDP and either isofagomine (cyan, PDB ID: 7LOU) or noeuromycin (gray, PDB ID: 7LOV). Panel **b** and **c** show the omit density map (Fo−Fc) of UDP-isofagonine (panel B, PDB ID: 7LOU) and UDP-noeuromycin (panel C, PDB ID: 7LOV) bound to the active site of TcdB-GTD. The omit map was calculated after omit refinement by REFMAC5, leaving out the active site inhibitor. The contour levels are at 3.0 σ.

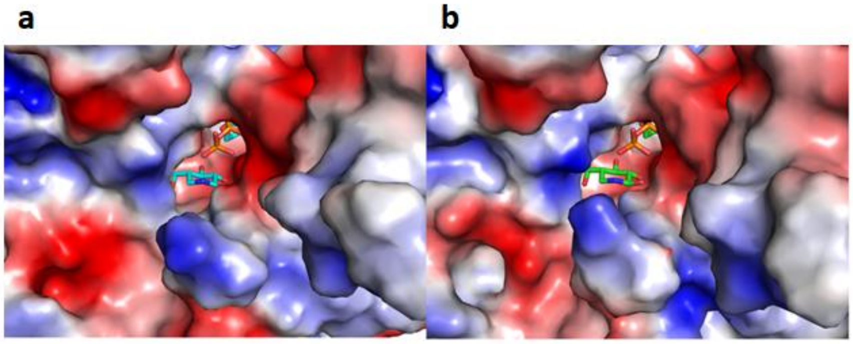


**Supplementary Figure 6.** Electrostatic surface representation of **a** the TcdB-GTD catalytic site bound with UDP and isofagomine (PDB ID:7LOU) and **b** UDP and noeuromycin (PDB ID: 7LOV). In the noeuromycin bound structure (panel B), the 2-hydroxyl points towards more negative charge pocket which could be responsible for the weaker binding of this inhibitor.

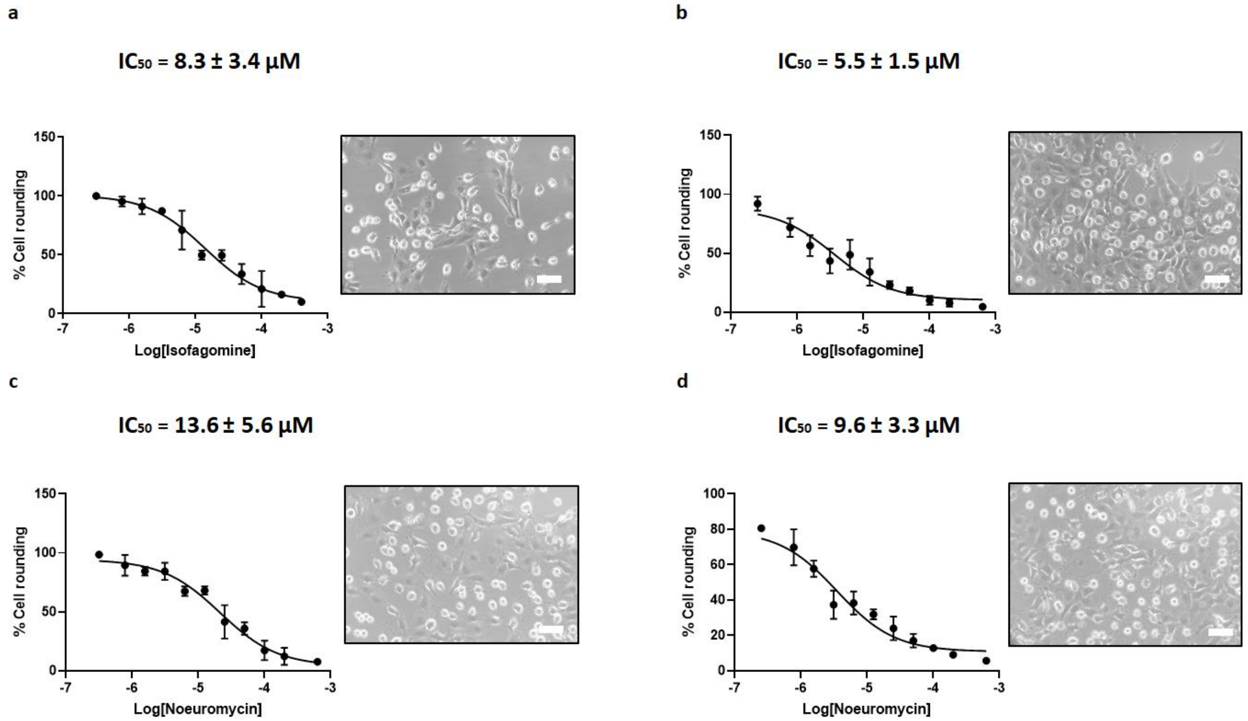


**Supplementary Figure 7. Efficacy of isofagomine and noeuromycin against Tcd toxin induced cell rounding.** Vero cells were pre-treated with varying concentrations of isofagomine or noeuromycin for 20 minutes before addition of 1 nM TcdA or 1 pM TcdB. Cells were incubated with TcdB or TcdA for 1.5 and 2 hr respectively before sample imaging. Rounded cells were quantified and expressed as a percentage of total cells. The $IC_{50}$ for each iminosugar was calculated by fitting the data to a non-linear regression model of Log[inhibitor] vs response. A representative plot from 1 experiment is shown on the left, with representative image of 12.5 µM iminosugar shown on the right (Scale bar represents 10µm). Data points represent the mean % cell rounding from 4 images of each well ± SEM. $IC_{50}$ values at the top of each graph represent the mean $IC_{50}$ ± SEM of 3 biological replicates (n=3). **a** TcdB + isofagomine. **b** TcdB + noeuromycin. **c** TcdA + isofagomine. **d** TcdA + noeuromycin. Source data provided as a Source Data file.

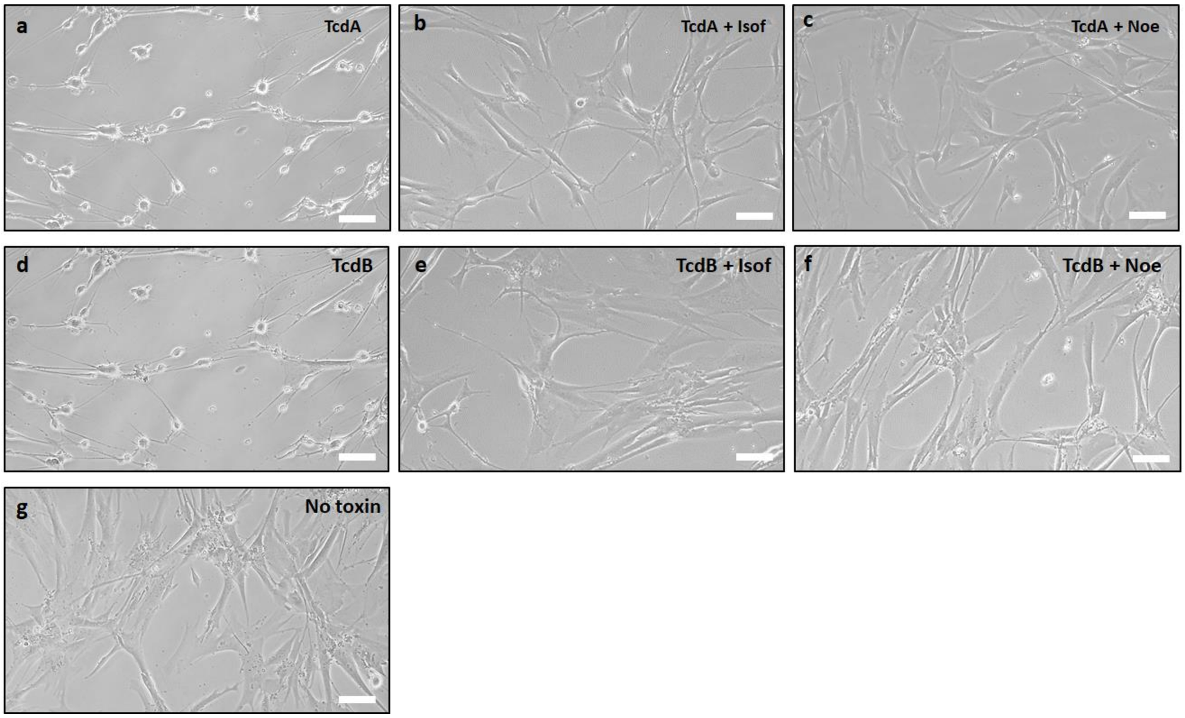


**Supplementary Figure 8. IMR90 cells treated with TcdA and TcdB and isofagomine or noeuromycin.** Representative images of human IMR90 cells from 3 experiments (n=3) . Scale bar represents 10µm. Cells were pre-treated with either 100 µM isofagomine (Isof) or noeuromycin (Noe) for 30 min before treatment with buffer, 1 nM TcdA or 0.1 nM TcdB and images were acquired after 6 hr. **a** TcdA treatment. **b** Treatment with isofagomine, then TcdA. **c** Treatment with noeuromycin, then TcdA. **d** TcdB treatment. **e** Treatment with isofagomine, then TcdB. **f** Treatment with noeuromycin, then TcdB. **g** Untreated control cells. Images show cell rounding with addition of TcdA or B versus no toxin control, and addition of inhibitors prevents cell rounding.

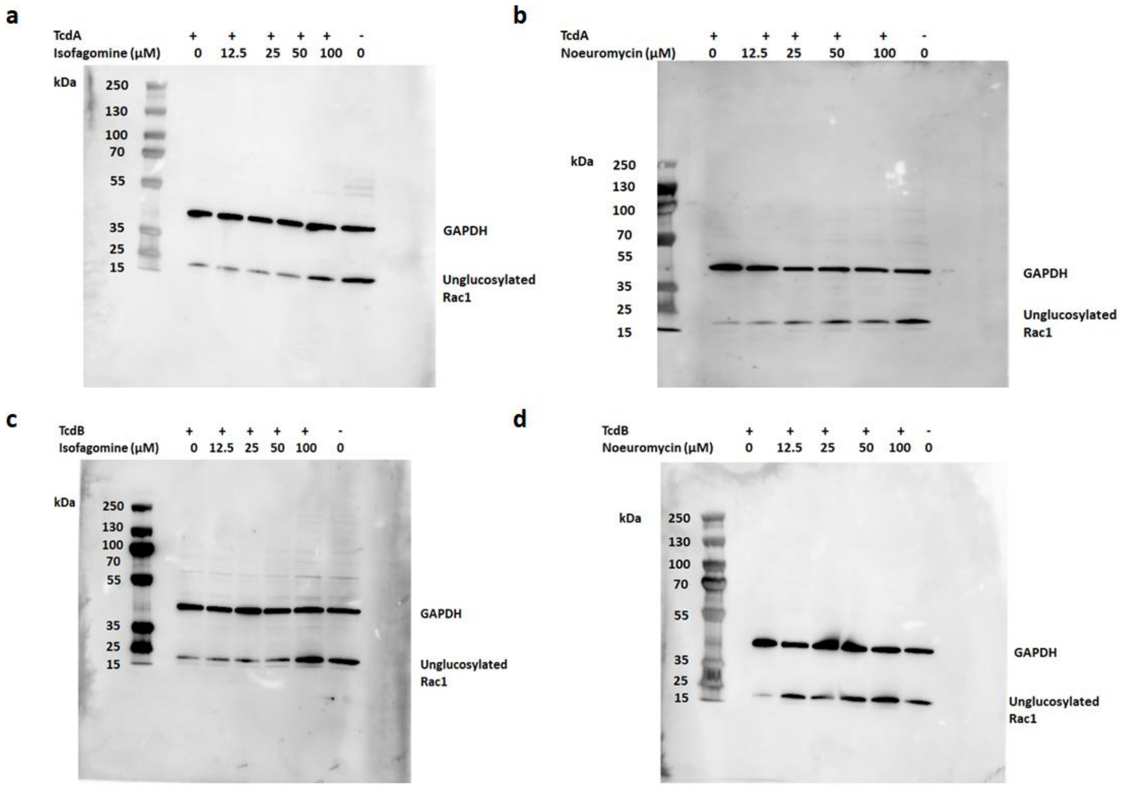


**Supplementary Figure 9. Raw Western blot analysis for Figure 6 of main text.** Representative western blots for intracellular Rac1 glucosylation (n=3). IMR90 cells were treated with either isofagomine or noeuromycin for 15 min, followed by treatment with either 1 nM TcdA or 0.1 nM TcdB. Cells were harvested as described in methods. Mab102 was used at 1/4000 dilution and recognizes un-glucosylated Rac1. Anti-GAPDH (1/8000) was used as a control for total protein levels. **a** TcdA and isofagomine treatment. **b** TcdA and noeuromycin treatment. **c** TcdB and isofagomine treatment. **d** TcdB and noeuromycin treatment.

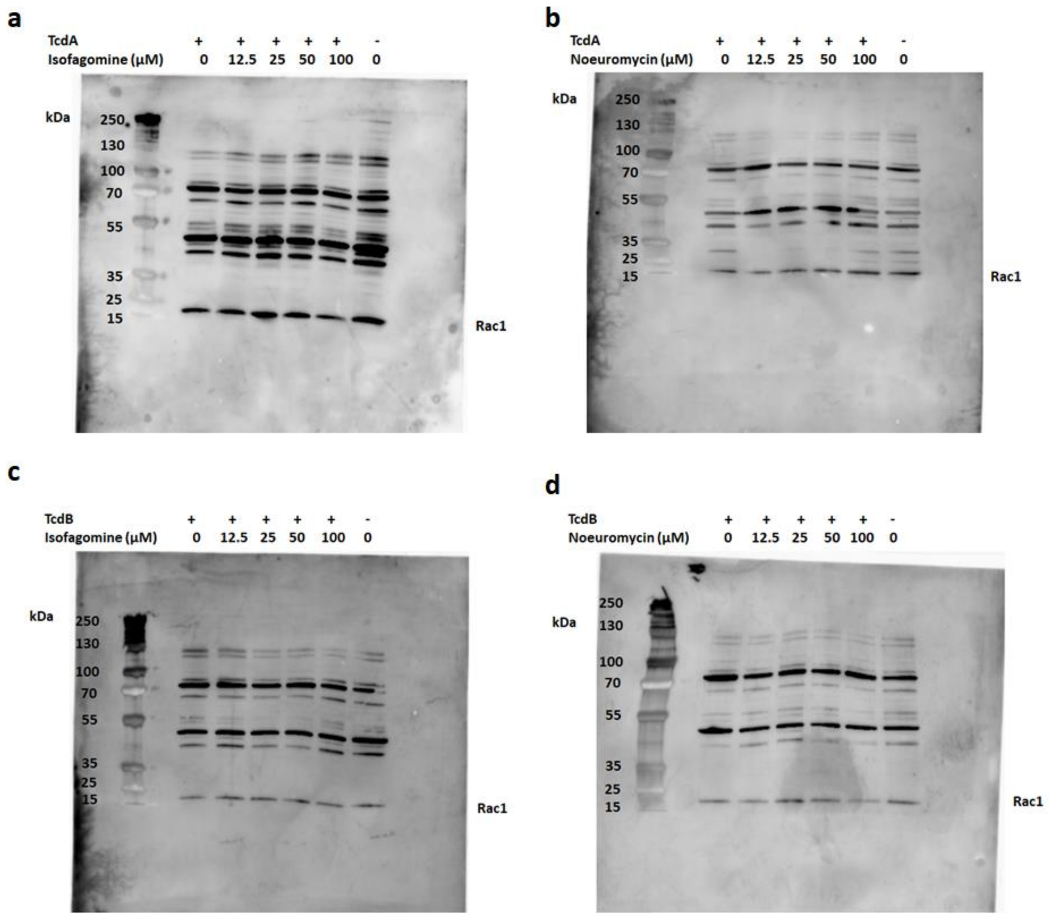


**Supplementary Figure 10. Raw Western blot analysis for Figure 6 of main text.** Representative Western blots for total Rac1 levels (n=3). IMR90 cells were treated with either Isofagomine or noeuromycin for 15 min, followed by treatment with either 1 nM TcdA or 0.1 nM TcdB. Cells were harvested as described in methods. Anti-Rac1 antibody (23A8) was used at 1/8000 dilution to control for total Rac1 levels. **a** TcdA and isofagomine treatment. **b** TcdA and noeuromycin treatment. **c** TcdB and isofagomine treatment. **d** TcdB and noeuromycin treatment.

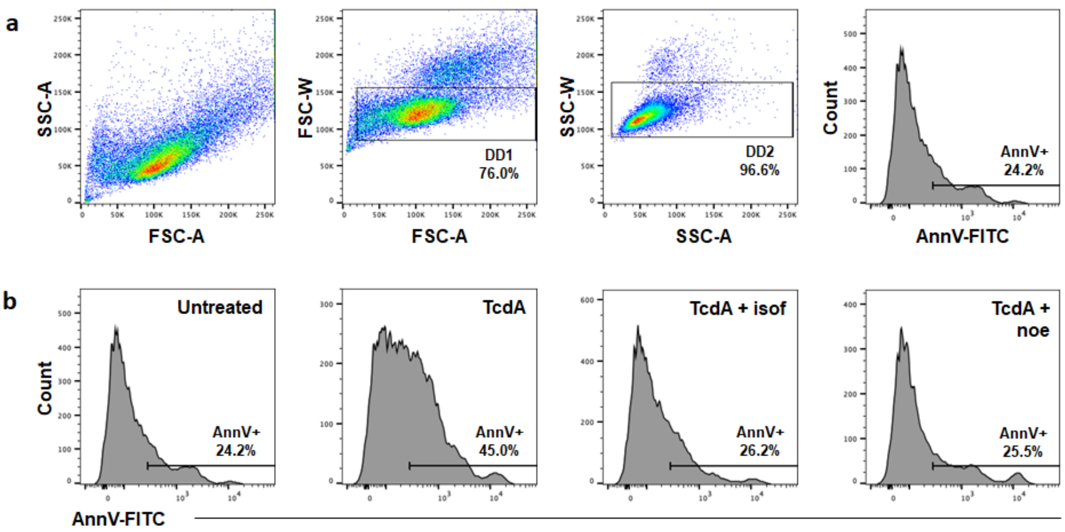


**Supplementary Figure 11. HT-29 cells treated with TcdA, isofagomine or noeuromycin and flow-cytometry analysis.** HT-29 Cells were pre-treated with isofagomine (isof; 500 µM) or noeuromycin (noe; 500 µM) for 30 min before addition of buffer or 1 nM TcdA. Cells were analyzed by flow cytometry after 24 hr. **a** Flow cytometry gating strategy to analyze single cells for AnnexinV positivity. **b** Representative flow cytometry plots of HT-29 cells treated with TcdA and iminosugars. Single cell events were gated for AnnexinV staining as a measure of apoptosis. Quantitation is shown in Figure 6c. DD is duplet discrimination.

**a**

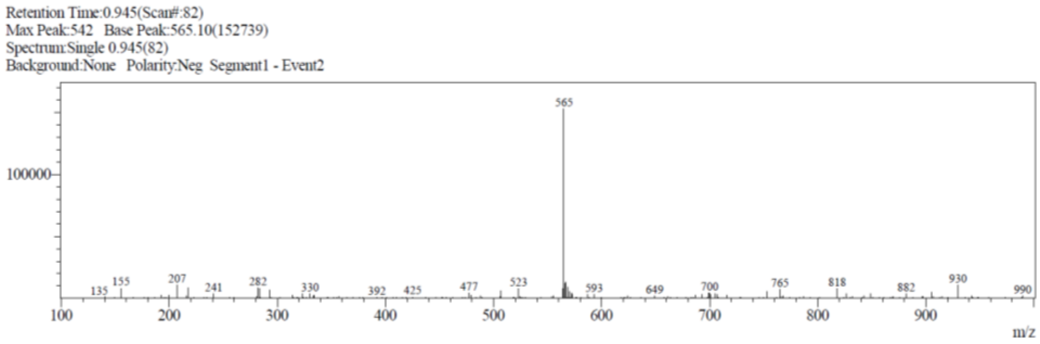


**b**

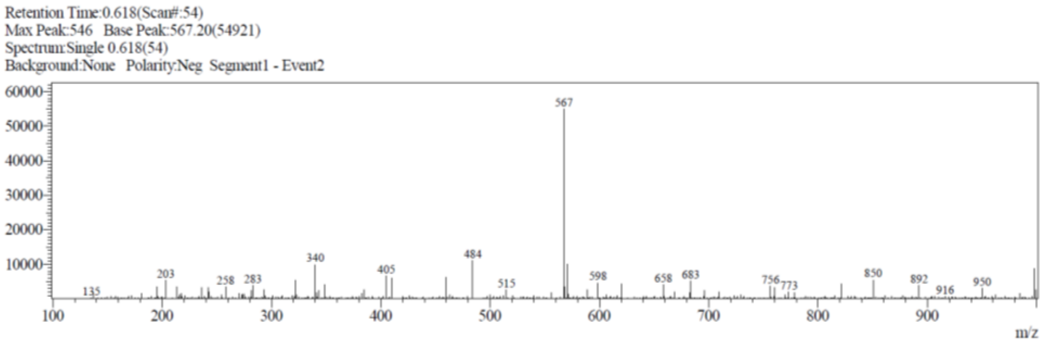


**Supplementary Figure 12. Mass spectrum of 1-$^{18}$O UDP-glucose.** To confirm enrichment of UDP-glucose with $^{18}$O a mass spectrum of 1-$^{18}$O UDP-glucose was acquired. **a** mass spectrum in negative mode of unlabeled UDP-glucose. Calculated mass of UDP-glucose [M – H ($C_{15}H_{24}N_2O_{17}P_2$)] was 566, mass found, 565. **b** mass spectrum in negative mode of 1-$^{18}$O UDP-glucose. Calculated mass of 1-$^{18}$O UDP-glucose [M – H ($C_{15}H_{24}N_2O_{17}P_2$)] was 568, mass found, 567.

## C. Supplementary Table 1

### Data collection and refinement statistics of TcDB-GTD-inhibitor structures.

| Dataset[a] | TcDB-GTD complex with isofagomine | TcDB-GTD complex with noeuromycin |
|---|---|---|
| **Unit cell data** | | |
| Space group | $P2_12_12_1$ | C2 |
| Cell parameters (Å, °) | a = 51.09, b = 121.34, c = 206.42<br>α = β = γ = 90.0 | a = 152.59, b = 82.68, c = 113.23<br>α = 90.0, β = 92.43, γ = 90.0 |
| $V_m$ (Å$^3$/Dalton) | 2.5 | 2.7 |
| Number of subunits in the asymmetric unit | 2.0 | 2.0 |
| **Data collection** | | |
| Beamline | LRL-CAT | AMX 17-ID-1 |
| Wavelength (Å) | 0.97931 | 0.97895 |
| Temperature (K) | 100 | 100 |
| Resolution range (Å) | 121.34 – 1.82 (1.85 – 1.82) | 28.96 – 2.50 (2.58 – 2.50) |
| Total number of observed reflections | 849285 (32989) | 341034 (31770) |
| Number of unique reflections | 115828 (5500) | 48863 (4458) |
| $R_{merge}$ (%)[b] | 6.9 (159.4) | 27.1 (178.5) |
| CC1/2 (%) | 99.9 (62.3) | 98.5 (49.8) |
| < I/σ(I)>[c] | 13.8 (1.0) | 6.2 (1.3) |
| Completeness (%) | 99.8 (97.8) | 99.9 (99.9) |
| Multiplicity | 7.3 (6.0) | 7.0 (7.1) |
| Wilson B-factor (Å$^2$) | 33.9 | 28.3 |
| **Refinement** | | |
| $R_{work}$ (%)[d] | 21.5 | 20.8 |
| $R_{free}$ (%)[e] | 25.2 | 24.8 |
| No. of atoms | 9489 | 9135 |
| Protein atoms | 8854 | 8808 |
| Ligand atoms | 70 | 72 |
| Solvent atoms | 565 | 255 |
| **Model quality** | | |
| **RMS deviation from ideal value** | | |
| Bond length (Å) | 0.007 | 0.003 |
| Bond angle (°) | 1.4 | 1.2 |
| **Average B-factor** | | |
| Protein atoms (Å$^2$) | 47.1 | 43.9 |
| Ligand atoms (Å$^2$) | 33.6 | 29.9 |
| Waters (Å$^2$) | 46.8 | 31.3 |
| **Ramachandran plot**[f] | | |
| Most favored regions (%) | 98.1 | 98.9 |
| Allowed regions (%) | 1.8 | 1.1 |
| Outlier regions (%) | 0.1 | 0.0 |
| PDB ID entry | 7LOU | 7LOV |

D. [a]Values in parentheses refer to the highest resolution shell.

E. [b]$R_{merge}$= $(\Sigma_{hkl}\Sigma_i|I_i(hkl) - < I(hkl)>|)/\Sigma_{hkl}\Sigma_i<I_i(hkl)>$, where $I_i(hkl)$ is the intensity of the $i^{th}$ measurement of reflection (hkl) and < I(hkl) > is its mean intensity.

F. [c]I is the integrated intensity and σ(I) is its estimated standard deviation.

G. [d]$R_{work}$ = $(\Sigma_{hkl}|Fo-Fc|)/\Sigma_{hkl}Fo$ where Fo and Fc are the observed and calculated structure factors.

H. [e]$R_{free}$ is calculated as for $R_{work}$ but from a randomly selected subset of the data (5%), which were excluded from the refinement calculation.

I. [f]Calculated by MOLPROBITY.

## D. Supplementary References


1. Andersch, J. & Bols, M. Efficient synthesis of isofagomine and noeuromycin. *Chemistry (Weinheim an der Bergstrasse, Germany)*. **7**, 3744-3747, (2001).
2. Liu, H. *et al*. Noeuromycin, a glycosyl cation mimic that strongly inhibits glycosidases. *J Am Chem Soc*. **123**, 5116-5117, (2001).
