# Supplementary material for: Inhibition of Clostridium difficile TcdA and TcdB toxins with transition state analogues
Source: Nat Commun. 2021 Nov 1;12:6285. doi: 10.1038/s41467-021-26580-6 (PMC8560925; doi:10.1038/s41467-021-26580-6)
Supplement: Supplementary file 3 — Reporting summary [file 41467_2021_26580_MOESM3_ESM.pdf]

nature research

Corresponding author(s): Vern Schramm

Last updated by author(s): Sep 30 2021

Reporting Summary

Nature Research wishes to improve the reproducibility of the work that we publish. This form provides structure for consistency and transparency in reporting. For further information on Nature Research policies, see our Editorial Policies and the Editorial Policy Checklist.

Statistics

For all statistical analyses, confirm that the following items are present in the figure legend, table legend, main text, or Methods section.

n/a	Confirmed	
☐	☒	The exact sample size (n) for each experimental group/condition, given as a discrete number and unit of measurement
☐	☒	A statement on whether measurements were taken from distinct samples or whether the same sample was measured repeatedly
☐	☒	The statistical test(s) used AND whether they are one- or two-sided<br>*Only common tests should be described solely by name; describe more complex techniques in the Methods section.*
☒	☐	A description of all covariates tested
☐	☒	A description of any assumptions or corrections, such as tests of normality and adjustment for multiple comparisons
☐	☒	A full description of the statistical parameters including central tendency (e.g. means) or other basic estimates (e.g. regression coefficient) AND variation (e.g. standard deviation) or associated estimates of uncertainty (e.g. confidence intervals)
☐	☒	For null hypothesis testing, the test statistic (e.g. F, t, r) with confidence intervals, effect sizes, degrees of freedom and P value noted<br>*Give P values as exact values whenever suitable.*
☒	☐	For Bayesian analysis, information on the choice of priors and Markov chain Monte Carlo settings
☒	☐	For hierarchical and complex designs, identification of the appropriate level for tests and full reporting of outcomes
☒	☐	Estimates of effect sizes (e.g. Cohen's d, Pearson's r), indicating how they were calculated

Our web collection on statistics for biologists contains articles on many of the points above.

Software and code

Policy information about availability of computer code

Data collection	Malvern Microcal PEAQ ITC control software, BD FACSDiva software used to run flow cytometer and data acquisition.
Data analysis	GraphPad Prism 8, Malvern Microcal PEAQ ITC analysis software, SigmaPlot version 6, iMOSFLM (version 7.2.2) , CCP4 programs (AIMLESS, BAVERAGE, SFCHECK, PHASER, REFMAC5), MolProbity, PyMOL, PHENIX (XTRIAGE), COOT, FIJI/Image J, FlowJo

For manuscripts utilizing custom algorithms or software that are central to the research but not yet described in published literature, software must be made available to editors and reviewers. We strongly encourage code deposition in a community repository (e.g. GitHub). See the Nature Research guidelines for submitting code & software for further information.

Data

Policy information about availability of data

All manuscripts must include a data availability statement. This statement should provide the following information, where applicable:

- Accession codes, unique identifiers, or web links for publicly available datasets
- A list of figures that have associated raw data
- A description of any restrictions on data availability

PDB Accesion Codes (7LOU, 7LOV). All figures have associated raw data.

Field-specific reporting

April 2020

Life sciences study design

All studies must disclose on these points even when the disclosure is negative.

Sample size	No sample size calculation was performed. Sample sizes are sufficient for this study and are similar to other published work which is referenced in the manuscript.
Data exclusions	None
Replication	at least 3, unless otherwise indicated. All attempts were successful.
Randomization	Not relevant to this study. Replicates provided randonmization.
Blinding	Not relevant to this study. All samples were experimental.

Reporting for specific materials, systems and methods

We require information from authors about some types of materials, experimental systems and methods used in many studies. Here, indicate whether each material, system or method listed is relevant to your study. If you are not sure if a list item applies to your research, read the appropriate section before selecting a response.

Materials & experimental systems

n/a	Involved in the study	
☐	☒	Antibodies
☐	☒	Eukaryotic cell lines
☒	☐	Palaeontology and archaeology
☒	☐	Animals and other organisms
☒	☐	Human research participants
☒	☐	Clinical data
☒	☐	Dual use research of concern

Methods

n/a	Involved in the study	
☒	☐	ChIP-seq
☐	☒	Flow cytometry
☒	☐	MRI-based neuroimaging

Antibodies

Antibodies used	Anti-Rac1 antibody Mab102 was from BD Biosciences (Mississauga, ON); Catalogue number 610650. Lot number 9140855.Total Rac1 levels were measured using mAb23A8 (Abcam); clone 23A8; Cat #Ab33186. Lot number 3526778. Loading controls using Anti-GAPDH antibody clone 6C5, catalog #ab8245. Lot number GR3380498-1 HRP Goat Anti-Mouse Ig secondary antibody from BD Biosciences (Catalogue number 554002). Lot number 0072774
Validation	See manufacturers websites. As stated on manufacturers websites. All antibodies used in this study are routinely tested by manufacturers.

Eukaryotic cell lines

Policy information about cell lines

Cell line source(s)	IMR90 (CCL-186), CHO-K1 (CCL61), Vero cells (CCL-81) and HT-29 (HTB-38) cells were all purchased from ATCC (American Type Culture Collection).
Authentication	Cell lines were authenticated by ATCC. Certificate of analysis is provided on ATCC website. STR profiling was performed.
Mycoplasma contamination	Cell lines were confirmed by ATCC to be free of mycoplasma contamination. Certificate analysis is provided on ATCC website.
Commonly misidentified lines (See ICLAC register)	None

Flow Cytometry

Plots

Confirm that:

- [x] The axis labels state the marker and fluorochrome used (e.g. CD4-FITC).
- [x] The axis scales are clearly visible. Include numbers along axes only for bottom left plot of group (a 'group' is an analysis of identical markers).
- [x] All plots are contour plots with outliers or pseudocolor plots.
- [x] A numerical value for number of cells or percentage (with statistics) is provided.

Methodology

Sample preparation	HT-29 cells were grown in McCoys 5A media supplemented with 10% FBS. After treatment cells were harvested using 0.25% trypsin, filtered through 0.22 um cell strainer caps into 5 mL polystyrene tubes and pelleted by centrifugation (1500 rpm 5 min). Cells were stained for AnnexinV-FITC as per the manufacturers instructions.
Instrument	LSRII Flow Cytometer (Becton Dickson)
Software	BD FACSDiva software used to run flow cytometer and data acquisition; BD FlowJo version 10 for data analysis and plot generation
Cell population abundance	300,000 cells were seeded per well of 6-well plate. After harvesting cells, 30,000 events (approx 10% total cells plated) were collected on flow cytometer for each condition.
Gating strategy	Total events > doublet discrimination (FSC-W vs FSC-A, SSC-W vs SSC-A) > Annexin V histogram

- [x] Tick this box to confirm that a figure exemplifying the gating strategy is provided in the Supplementary Information.
